# Anomalous blueshift of aperture resonance enabled by the loss of a thin film

**DOI:** 10.1038/s41598-020-79224-y

**Published:** 2020-12-16

**Authors:** Jisoo Kyoung

**Affiliations:** Department of Physics, Dankook University, 119 Dandae-ro, Dongnam-gu, Cheonan-si, Chungnam 31116 Korea

**Keywords:** Nanophotonics and plasmonics, Sub-wavelength optics, Terahertz optics, Metamaterials

## Abstract

The substrate effects on aperture resonance have been widely studied because the resonance peak position is key for sensing, communications, and field enhancement applications. So far, the theoretical works have focused on the lossless infinite substrate case, which only explains the resonance peak redshift by the substrate index. The loss effect has not been investigated yet because lossy infinite substrate significantly reduces the aperture transmission. Therefore, this work studied the loss effect on aperture resonance via an analytic model of the transmission though slot antennas on a finite substrate. When the substrate thickness was very thin compared to the wavelength, the transmittance remained high even for a large imaginary part of the refractive index; furthermore, the developed model predicted a strong blueshift when this imaginary part exceeds the real part. Besides, the zero of the imaginary part of the coupling strength was crucial in determining the resonance frequency for both infinite and finite substrates. Thus, this study can contribute to the material characterization, label-free detection, and efficient design of active metamaterials.

## Introduction

**Figure 1:**
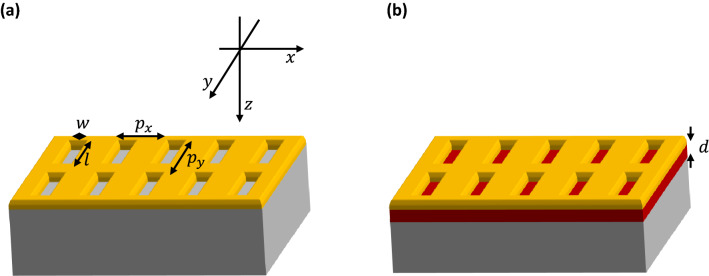
Schematics of typical (**a**) three-layer (superstrate/metal slot antenna array/infinite substrate) and (**b**) four-layer (superstrate/metal slot antenna array/thin film/infinite substrate) systems.

The transmission properties of subwavelength holes in metal films have been extensively studied because of the extraordinary optical transmission phenomena involved^1–5^. Among these apertures, the slot antennas, based on a narrow rectangular hole, are drawing much attention since they strongly enhance the electric field^6–10^. They also exhibit a strong resonance transmission peak. For free-standing devices, the wavelength of this peak is about twice the length of the antenna, like in ordinary dipole antennas^4,9,11–13^; Choe et al. recently defined the slot antennas as bound charge oscillators having a dipole radiation pattern^14^. Since the dielectric surroundings significantly influence the optical properties of subwavelength holes, the resonance peak of a slot antenna can be shifted due to its substrate characterastics^4,15^. The THz frequency range is appropriate to investigate such substrate effects because most metals are perfect conductors and various substrates such as silicon, glass, quartz, and GaP are dispersionless and lossless. Many experiments and simulations have demonstrated strong redshifts by the substrate^12,16–20^. Rigorous analytic calculations have proved that the resonance peak is located around the zero of the imaginary part of the averaged coupling strength and can be simply expressed as^14^

1 $$\frac{{\uplambda }_{\mathrm{res}}}{l}=\sqrt{2({n}_{\mathrm{s}}^{2}+1)} ,$$

where ${\uplambda }_{\mathrm{res}}$ is the resonance wavelength, $l$ is the slot antenna length, and ${n}_{\mathrm{s}}$ is the refractive index of the substrate. According to Eq. (1), the increase of ${n}_{\mathrm{s}}$ results in a redshift. This calculation was recently extended to both circular and annular holes^12^. So far, only lossless substrates with infinite thickness have been considered (Fig. 1a). Nowadays, however, thin dielectric layers are often inserted between the substrate and the metal nanostructure to actively modulate the transmission or reflection properties (Fig. 1b)^21–27^. Therefore, the theoretical calculations must be adjusted to correctly describe also the effect of such thin active films on the aperture resonance. In this work, previous theoretical studies were extended to consider thin films having a complex refractive index. When the real part of their refractive index increases, the redshift of the aperture resonance was still observed, but its peak shape quite differed from the infinite substrate case. Moreover, the resonance peak could be blueshifted when incrementing the thin film loss, unlike for infinite substrates.

## Theoretical model

A three-layer model (superstrate/slot antenna array/substrate) was previously used to investigate the substrate effects (Fig. 1a)^20,28^. By adding a thin active layer between the slot antenna array and the substrate, a four-layer system (superstrate/slot antenna array/thin film/substrate) is obtained (Fig. 1b). Hereafter, the width, length, thickness, and period in the x and y directions of the metal slot antenna array are denoted by $w, l, h, {p}_{x}$, and ${p}_{y}$ respectively, while $d$ represents the thin film thickness; for simplicity, the refractive indices of the superstrate and inside the aperture were assumed to be unity. The incident wave polarized along the x direction can be described by the magnetic field as follows:

2 $${H}_{y}^{I, \mathrm{inc}}=\sqrt{\frac{{\varepsilon }_{0}}{{\mu }_{0}}}{e}^{ik\left(z+h\right)},$$

where $k=\frac{2\pi }{\lambda }$ and the time dependence term ${e}^{-i\omega t}$ is omitted. The corresponding electric field can be derived from the Maxwell equation:

3 $$\overrightarrow{\nabla }\times \overrightarrow{H}=-i\omega \varepsilon \overrightarrow{E}$$

where $\varepsilon$ is the permittivity. Although the incident wave has only the y component of the magnetic field, the reflection wave can have three components, and each of them is decomposed via modal expansion:

4 $${\overrightarrow{H}}^{I,\mathrm{ref}}=\sqrt{\frac{{\varepsilon }_{0}}{{\mu }_{0}}}{\sum }_{m,n}{\overrightarrow{R}}_{mn}{e}^{-i\left({k}_{m}\left(x-w/2\right)+{k}_{n}\left(y-l/2\right)+{\kappa }_{mn}\left(z+h\right)\right)},$$

where ${k}_{m}=\frac{2\pi }{{p}_{x}}m,{k}_{n}=\frac{2\pi }{{p}_{y}}n$, and ${\kappa }_{mn}=\sqrt{{k}^{2}-{k}_{m}^{2}-{k}_{n}^{2}}$. Inside the slot antenna, the single mode approximation can be applied since each slot works as a bound charge oscillator^14^:

5 $${\overrightarrow{E}}^{II}=\widehat{x}\mathrm{sin}\left(\frac{\pi y}{l}\right)\left[A{e}^{i\beta z}+B{e}^{-i\beta z}\right]$$

where $\beta =\sqrt{{k}^{2}-{\left(\frac{\pi }{l}\right)}^{2}}$, and $A$ and $B$ are forward and backward propagation coefficients, respectively. Likewise, in the thin film, both the forward and backward propagations exist which can be written as:

6 $${\overrightarrow{H}}^{III}=\sqrt{\frac{{\varepsilon }_{0}}{{\mu }_{0}}}{\sum }_{m,n}\left({\overrightarrow{C}}_{mn}{e}^{i\left({\kappa }_{mn}^{{n}_{t}}z\right)}+{\overrightarrow{D}}_{mn}{e}^{-i\left({\kappa }_{mn}^{{n}_{t}}z\right)}\right){e}^{-i\left({k}_{m}\left(x-w/2\right)+{k}_{n}\left(y-l/2\right)\right)}$$

where ${\kappa }_{mn}^{{n}_{t}}=\sqrt{{n}_{t}^{2}{k}^{2}-{k}_{m}^{2}-{k}_{n}^{2}}$ , ${n}_{t}$ is the refractive index of the thin film, and ${\overrightarrow{C}}_{mn}$ and ${\overrightarrow{D}}_{mn}$ are the portions of the forward and backward radiation, respectively, at each mode inside the thin film. In the substrate, only the forward transmission exists:

7 $${\overrightarrow{H}}^{IV}=\sqrt{\frac{{\varepsilon }_{0}}{{\mu }_{0}}}{\sum }_{m,n}{\overrightarrow{T}}_{mn}{e}^{-i\left({k}_{m}\left(x-w/2\right)+{k}_{n}\left(y-l/2\right)-{\kappa }_{mn}^{{n}_{s}}\left(z-d\right)\right)}.$$

After applying the boundary conditions at the three interfaces (superstrate/metal slot antenna array, metal slot antenna array/thin film, thin film/substrate) and assuming $\lambda \gg h$, the transmission coefficient of the four-layer system ${T}_{4}$ can be expressed as

8 $${T}_{4}=\frac{{n}_{t}^{2}}{{n}_{s}}{\left(\frac{4}{\pi }\right)}^{4}{\left(\frac{wl}{{p}_{x}{p}_{y}}\right)}^{2}\frac{1}{{\left|{W}_{1}+{W}_{4}\right|}^{2}}\frac{1}{{\left|{F}_{00}^{y}\right|}^{2}} ,$$

where

9 $${W}_{1}=\frac{1}{2}\left(\frac{wl}{{p}_{x}{p}_{y}}\right){\sum }_{m,n}\frac{\left({k}^{2}-{k}_{n}^{2}\right)}{k{\kappa }_{mn}}{J}_{mn}^{2},$$

10 $${W}_{4}=\frac{1}{2}\left(\frac{wl}{{p}_{x}{p}_{y}}\right){\sum }_{m,n}\frac{{E}_{mn}^{y}}{{F}_{mn}^{y}}\frac{\left({n}_{t}^{2}{k}^{2}-{k}_{n}^{2}\right)}{k{\kappa }_{mn}^{{n}_{t}}}{J}_{mn}^{2},$$

11 $${J}_{mn}={\text{sinc}}\left(\frac{w{k}_{m}}{2}\right)\left[{\text{sinc}}\left(\frac{\pi }{2}+\frac{l{k}_{n}}{2}\right)+{\text{sinc}}\left(\frac{\pi }{2}-\frac{l{k}_{n}}{2}\right)\right],$$

12 $$\left({C}_{mn}^{y}+{D}_{mn}^{y}\right)={E}_{mn}^{y}{T}_{mn}^{y} ,$$

and

13 $$\left({C}_{mn}^{y}-{D}_{mn}^{y}\right)={F}_{mn}^{y}{T}_{mn}^{y}.$$

The three-layer system can be easily recovered when setting ${n}_{t}={n}_{s}$ in the above calculations; in that case, ${\overrightarrow{D}}_{mn}=0$ and ${E}_{mn}^{y}={F}_{mn}^{y}=1$ because the thin film/substrate interface disappears. Therefore, the explicit form of the transmission coefficient of this model (${T}_{3}$) is:

14 $${T}_{3}={n}_{s}{\left(\frac{4}{\pi }\right)}^{4}{\left(\frac{wl}{{p}_{x}{p}_{y}}\right)}^{2}\frac{1}{{\left|{W}_{1}+{W}_{3}\right|}^{2}} ,$$

where

15 $${W}_{3}=\frac{1}{2}\left(\frac{wl}{{p}_{x}{p}_{y}}\right){\sum }_{m,n}\frac{\left({n}_{s}^{2}{k}^{2}-{k}_{n}^{2}\right)}{k{\kappa }_{mn}^{{n}_{s}}}{J}_{mn}^{2}.$$

## Three-layer system

**Figure 2:**
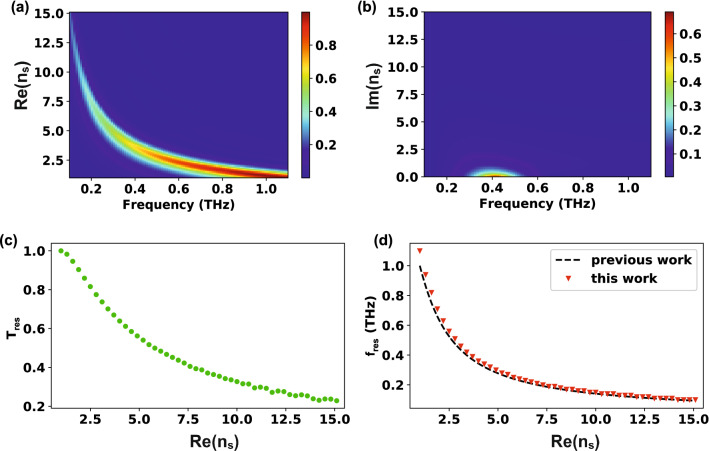
Transmission characteristics of three-layer system. The geometry of the slot antennas: $w=25\,\upmu {\mathrm{m}}, l=150\,\upmu {\mathrm{m}}, h=70\, \mathrm{nm}, {p}_{x}=127\,\upmu {\mathrm{m}},$ and ${p}_{y}=180\,\upmu {\mathrm{m}}$. (**a**) Transmission as functions of frequency and real part ($\mathrm{Re}({n}_{s})$) of refractive index of the substrate. (**b**) Transmission as functions of frequency and imaginary part ($\mathrm{Im}({n}_{s})$) of refractive index of the substrate. (**c**) Maximum transmission versus $\mathrm{Re}({n}_{s})$. (**d**) Resonance frequency versus $\mathrm{Re}({n}_{s})$. The downward triangles and dotted line represents the calculation results of the proposed model and previously reported equations (Eq. 1), respectively^14^.

For validation, first, the transmission spectra of a typical three-layer system were calculated by using Eq. (8) with ${n}_{t}={n}_{s}$ and compared them with previous works. The calculation was performed under the following conditions: $w=25\,\upmu {\mathrm{m}}, l=150\,\upmu {\mathrm{m}}, h=70\, \mathrm{nm}, {p}_{x}=127\,\upmu {\mathrm{m}},$ and ${p}_{y}=180\,\upmu {\mathrm{m}}$. $70\, \mathrm{nm }(h)$ is a sufficient thickness to consider a real metal such as gold as a perfect conductor (see the supplementary information). The transmission as functions of the real part of ${n}_{s}$ ($\mathrm{Re}({n}_{s})$) and the frequency was plotted in Fig. 2a. When ${n}_{s}$ is small ($\approx 1$), the resonance peak is around 1 THz (300-$\upmu {\mathrm{m}}$ wavelength), which is almost twice the slot antenna length. As mentioned in Section I, this is typical of free-standing slot antennas similar to dipole antennas. When ${n}_{s}$ increases, the resonance peak shifts toward shorter frequencies (longer wavelengths) with decreasing the transmission. To better understand the ${n}_{s}$ effect, also the maximum transmission and resonance frequency were plotted as functions of $\mathrm{Re}({n}_{s})$ (Fig. 2c, d), observing that the maximum transmission (green circles in Fig. 2c) gradually decreases as ${n}_{s}$ increases. This is mainly due to the impedance mismatch between the slot antennas and the substrate^20^. Moreover, the resonance frequency (red downward triangles in Fig. 2d) undergoes a strong redshift as ${n}_{s}$ increases, consistently with the previous reports^19,20,29^. The analytic form of the resonance frequency (Eq. 1), reported in previous work^28^, can be derived by imposing the zero of the imaginary part of coupling strength ($\mathrm{Im }\left({W}_{1}+{W}_{3}\right)=0$) in Eq. (14). As clearly seen, the resonance position calculated from our model (red downward triangle in Fig. 2d) and from Eq. (1) (black dotted line in Fig. 2d) are remarkably well-matched showing the validity of our calculation. We also performed the full-wave simulation using FDTD (finite difference time domain) method and confirmed that two results from our calculations and the FDTD are consistent (see the supplementary information).

Unlike the $\mathrm{Re}({n}_{s})$ influence, the effect of the imaginary part of ${n}_{s}$ ($\mathrm{Im}\left({n}_{s}\right)$) on aperture resonance has been rarely studied for infinite substrates. This is because the substrate loss is exponentially proportional to $\mathrm{Im}\left({n}_{s}\right)$; namely, even if $\mathrm{Im}\left({n}_{s}\right)$ is not very large, the transmittance becomes very small and the resonance disappears. Figure 2b illustrates qualitatively the substrate loss effect on the transmission. When increasing $\mathrm{Im}({n}_{s})$, the transmission rapidly decreases and finally the resonance disappears when $\mathrm{Im}\left({n}_{s}\right)$ is over unity. Therefore, the three-layer system is not appropriate to investigate the $\mathrm{Im}\left({n}_{s}\right)$ effect on aperture resonance, unlike the four-layer model, where the transmittance value remains high even if $\mathrm{Im}\left({n}_{s}\right)$ becomes very large, as long as the thickness of the thin film ($d$) is considerably smaller than the wavelength.

## Four-layer system

**Figure 3:**
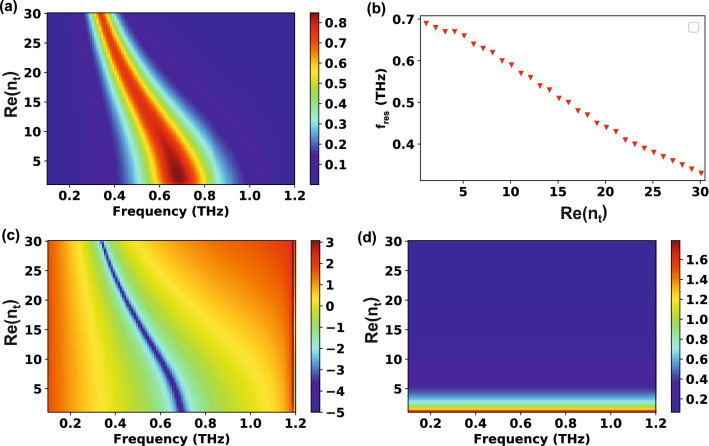
Effect of the real part of the thin film refractive index ($\mathrm{Re}({n}_{t})$) on the transmission characteristics of a four-layer system. The geometry of the slot antennas: $w=25\,\upmu {\mathrm{m}}, l=150\,\upmu {\mathrm{m}}, h=70\, \mathrm{nm}, {p}_{x}=127\,\upmu {\mathrm{m}},$ ${p}_{y}=180\,\upmu {\mathrm{m}}$ and $d=240\, \mathrm{nm}$. The refractive index of the substrate is fixed at 2.0. (**a**) Transmission as functions of frequency and $\mathrm{Re}({n}_{t})$. (**b**) Relationship between the resonance frequency and $\mathrm{Re}({n}_{t})$. $\mathrm{Re}({n}_{t})$ versus (**c**) $\mathrm{log}(\mathrm{Im }\left({W}_{1}+{W}_{3}\right))$ and (**d**) $1/\left|{F}_{00}^{y}\right|$.

Next, the proposed theoretical model was applied to a four-layer system. In this case, ${n}_{s}$ was fixed (2.0), while the real and imaginary parts of ${n}_{t}$ ($\mathrm{Re}({n}_{t})$ and $\mathrm{Im}\left({n}_{t}\right)$, respectively) were varied. The thickness of the thin film ($d$) was $240\, \mathrm{nm}$ ($\sim\uplambda /1000$). The transmission versus frequency and $\mathrm{Re}({n}_{t})$ was drawn in Fig. 3a. As in the three-layer system, the resonance peak is redshifted when increasing $\mathrm{Re}({n}_{t})$. However, there are several differences between the three- and four-layer cases. First, the transmission through the slot antennas is 70% or higher even at very large $\mathrm{Re}({n}_{t})$; this is because the overall thickness ($\sim 300\, \mathrm{nm}$) of device is very thin compared to the wavelength (~ $300\mathrm{ \mu m}$). Second, the trend of this red shift is nearly linear (Fig. 3b) for the four-layer system, while it follows a $1/\mathrm{n}$ trend for the three-layer one (Fig. 2d). Moreover, the figure-of-merit of the peak shift ($\Delta \mathrm{f}/\Delta \mathrm{n})$ is much larger for the three-layer system (~ 0.07 THz) than for the four-layer one (~ 0.01 THz). These differences might be due to the finite thickness of the thin film.

As mentioned before, the resonance peak of a tree-layer system is determined by the zero of the imaginary part of the coupling strength ($\mathrm{Im }\left({W}_{1}+{W}_{3}\right)=0$), which is in the denominator of Eq. (14). For a four-layer system, instead, the two terms of the denominator (Eq. 8), that is, ${\left|{W}_{1}+{W}_{4}\right|}^{2}$ and ${\left|{F}_{00}^{y}\right|}^{2}$, compete; to verify which of them is dominant for the resonance frequency determination, both $\mathrm{log}(\mathrm{Im }\left({W}_{1}+{W}_{3}\right))$ and $1/\left|{F}_{00}^{y}\right|$ were plotted (Fig. 3c, d). Since ${F}_{mn}^{y}$ is defined by Eq. (13), ${F}_{mn}^{y}$ can be considered a zero-order proportionality factor determining how many waves are transmitted at the thin film/substrate interface. The $\left|{F}_{00}^{y}\right|$ plot reveals a negligible frequency dependence, while there is strong correlation between the transmission (Fig. 3a) and $\mathrm{log}(\mathrm{Im }\left({W}_{1}+{W}_{3}\right))$ graph (Fig. 3c) plots. Therefore, the zero of the imaginary part of the coupling strength is crucial in determining the peak position for both three- and four-layer systems.

**Figure 4:**
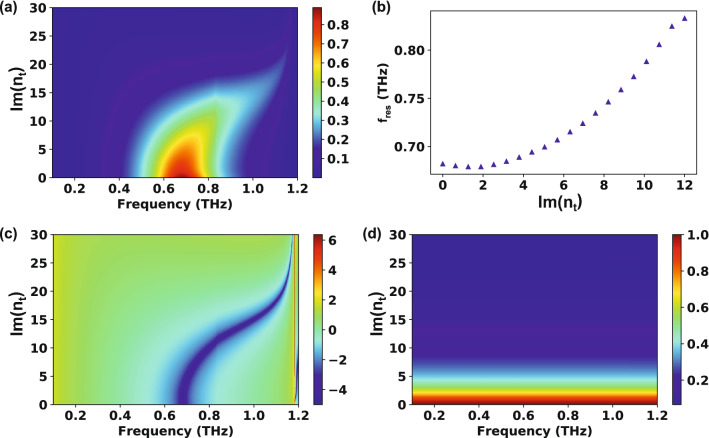
Effect of the imaginary part of the thin film refractive index ($\mathrm{Im}({n}_{t})$) on the transmission characteristics of a four-layer system. The geometry of the slot antennas: $w=25\,\upmu {\mathrm{m}}, l=150\,\upmu {\mathrm{m}}, h=70\, \mathrm{nm}, {p}_{x}=127\,\upmu {\mathrm{m}},$ ${p}_{y}=180\,\upmu {\mathrm{m}}$ and $d=240\, \mathrm{nm}$. The refractive index of the substrate is fixed at 2.0. (**a**) Transmission as functions of frequency and $\mathrm{Im}({n}_{t})$. (**b**) Relationship between the resonance frequency and $\mathrm{Im}({n}_{t})$. $\mathrm{Im}({n}_{t})$ versus (**c**) $\mathrm{log}(\mathrm{Im }\left({W}_{1}+{W}_{3}\right))$ and (**d**) $1/\left|{F}_{00}^{y}\right|$.

Since the thin film thickness is much lower than the wavelength, the $\mathrm{Im}({n}_{t})$ effect can also be investigated; $\mathrm{Re}({n}_{t})$ was fixed at 2.0. The transmission as functions of frequency and $\mathrm{Im}({n}_{t})$ is displayed in Fig. 4a. Apart from the three-layer system, the transmission peak survives until $\mathrm{Im}({n}_{t})$ reaches about 12. To verify the blueshift clearly, Fig. 4b plots $\mathrm{Im}({n}_{t})$ against the peak frequency. When $\mathrm{Im}({n}_{t})$ is smaller than $\mathrm{Re}({n}_{t})$, the resonance position hardly changes even if $\mathrm{Im}({n}_{t})$ increases. On the contrary, when $\mathrm{Im}({n}_{t})$ becomes larger than $\mathrm{Re}({n}_{t})$, a strong blueshift occurs. These unique properties can be used to investigate metal insulator phase transitions because the imaginary and real parts intersect in such events^30^. For example, during the phase transition of vanadium dioxide (VO_2_) thin film, there are two steps: first, the real part of the index largely increases and then the imaginary part largely increases and overwhelms the real part^31^. However, such two-step transition has not been clearly distinguished by just monitoring the transmission amplitude; only monotonic decrease of the transmission has been observed^18,32,33^. Instead, since the red or blue shifts are determined according to the relative size between the real part and the imaginary part, the two steps of phase transition can be distinguished by tracking the peak position (manuscript being prepared). The $\mathrm{Im}({n}_{t})$ relationships with $\mathrm{log}(\mathrm{Im }\left({W}_{1}+{W}_{3}\right))$ and $1/\left|{F}_{00}^{y}\right|$ were plotted as well (Fig. 4c, d); also in this case, the $1/\left|{F}_{00}^{y}\right|$ shows no frequency dependency, while the zero of $\mathrm{Im }\left({W}_{1}+{W}_{3}\right)$ condition is dominant in determining the resonance peak position.

**Figure 5:**
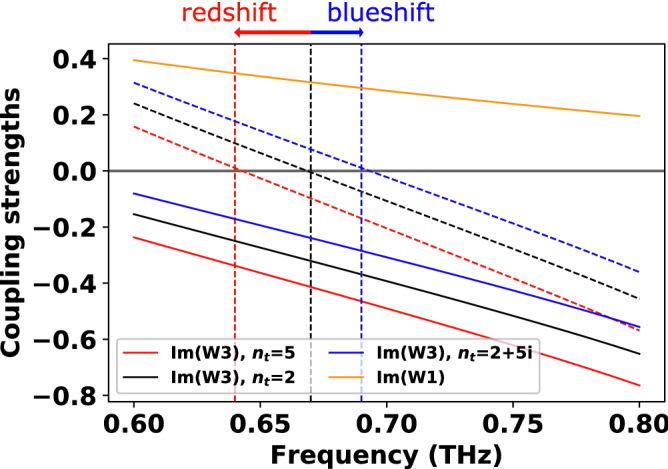
Plots of coupling strengths $\mathrm{Im }\left({W}_{1}\right)$ (orange solid line), $\mathrm{Im }\left({W}_{3}\right)$ (black, red, blue solid lines), and $\mathrm{Im }\left({W}_{1}+{W}_{3}\right)$ (black, red, blue dotted lines) for different refractive indices of thin film (${n}_{t}$). The black, red, and blue colors represent ${n}_{t}=2$, ${n}_{t}=5$, and ${n}_{t}=2+5i$, respectively. The resonance transmission positions (black, red, blue vertical dotted lines) occur around the zero-crossing points of each dotted line ($\mathrm{Im }\left({W}_{1}+{W}_{3}\right)$).

To further confirm the influence of the coupling strengths (${W}_{1}$ and ${W}_{3}$) on the resonant frequency, $\mathrm{Im }\left({W}_{1}\right)$,$\mathrm{Im }\left({W}_{3}\right)$, and $\mathrm{Im }\left({W}_{1}+{W}_{3}\right)$ were calculated as functions of the frequency at different ${n}_{t}$ (Fig. 5). Since $\mathrm{Im }\left({W}_{1}+{W}_{3}\right)$ is zero around the resonance peak, the track of the peak shift could be kept by investigating the zero-crossing property of $\mathrm{Im }\left({W}_{1}+{W}_{3}\right).$ As expressed in Eq. (9), $\mathrm{Im }\left({W}_{1}\right)$ is independent of ${n}_{t}$ and ${n}_{s}$ (orange solid line in Fig. 5) and monotonically decrease in the 0.6 to 0.8 THz spectral range with positive values. On the contrary, when ${n}_{t}={n}_{s}=2$, $\mathrm{Im }\left({W}_{3}\right)$ has negative values (black solid line in Fig. 5) and the zero-crossing of $\mathrm{Im }\left({W}_{1}+{W}_{3}\right)$ occurs at 0.67 THz (vertical black dotted line in Fig. 5). When $\mathrm{Re}({n}_{t})$ increases to 5, $\mathrm{Im }\left({W}_{3}\right)$ consequently decreases almost uniformly (red solid line in Fig. 5) so that the zero-crossing of $\mathrm{Im }\left({W}_{1}+{W}_{3}\right)$ (vertical red dotted line) is at a lower frequency (0.64 THz), resulting in the resonance redshift. In contrast, in the presence of the imaginary part (${n}_{t}=2+5i$), $\mathrm{Im }\left({W}_{3}\right)$ increase overall (blue solid line in Fig. 5) and, thereby, the zero-crossing is found at a higher frequency (0.69 THz), leading to the blueshift. There results indicate that the resonance frequency is redshifted (blueshifted) when the coupling strength between slot antennas and thin film decreases (increases).

**Figure 6:**
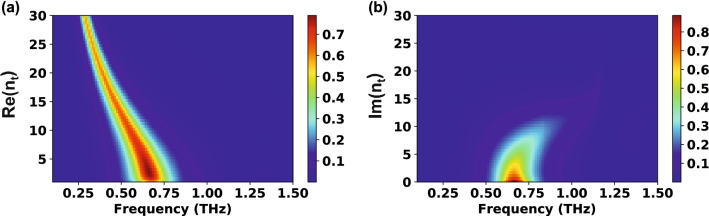
Transmission characteristics of four-layers system with narrower slots. The geometry of the slot antennas: $w=500\, \mathrm{nm}, l=150\,\upmu {\mathrm{m}}, h=70\, \mathrm{nm}, {p}_{x}=127\,\upmu {\mathrm{m}},$ ${p}_{y}=180\,\upmu {\mathrm{m}}$ and $d=240\, \mathrm{nm}$. The refractive index of the substrate is fixed at 2.0. (**a**) Transmission as functions of frequency and $\mathrm{Re}({n}_{t})$. (**b**) Transmission as functions of frequency and $\mathrm{Im}\left({n}_{t}\right)$.

We repeated the above calculations for a much narrower slot ($w=500\, \mathrm{nm}$); the resulting $\mathrm{Re}({n}_{t})$ and $\mathrm{Im}\left({n}_{t}\right)$ dependencies are illustrated in Fig. 6a, b, respectively. In this case, the resonance peak is much narrower compared to the wider slot case. This happens because the light scattering by the slot antennas follows quasi-Lorentzian trend and the line width is determined by the width of the slot antennas^14^. Although the spectral line width is different, the red- or blueshift characteristic is very similar between the wider and narrower slot antennas. Therefore, it can be concluded that such peak shifts are mainly determined by the material parameters of the thin film rather than the slot antenna geometries. To check the robustness of the peak shift, calculations are also performed at various thicknesses of the thin film. It weakens as the thickness decreases, but a peak shift is still observed even at 10 nm thickness (see the supplementary information).

## Conclusion

In conclusion, we have investigated thin film effect on aperture resonances both with and without loss. A novel theoretical model for the transmission through slot antenna array fabricated on the thin film/substrate structures (four-layer systems) was developed. Our model gave consistent results with the previous works when applied to the three-layer system. $\mathrm{Re}({n}_{t})$ increments make the resonance peak shifting toward longer wavelengths (redshift), while $\mathrm{Im}({n}_{t})$ increases induce a blueshift. Especially, the blueshift becomes significant when $\mathrm{Im}({n}_{t})$ exceeds $\mathrm{Re}({n}_{t})$, which allows us to characterize the properties of active materials. Recently, an active layer is presently inserted between the metal slot antenna array and their infinite substrates to modulate the transmission and reflection properties. Therefore, this theoretical work could help to design and understand the working principles of these active metamaterials^24,27,34^. Moreover, since the resonance frequency is very sensitive to the dielectric environment, the observation of its shift can be used for various chemical and biological sensing applications^35–37^. To test the feasibility of such applications, we additionally calculated the case where a thin film is now placed on the slot antenna and verified that the red and blue shifts could also be observed (see the supplementary information).

## Supplementary information

Supplementary Information
