# Supplementary material for: Anomalous blueshift of aperture resonance enabled by the loss of a thin film
Source: Sci Rep. 2020 Dec 16;10:22100. doi: 10.1038/s41598-020-79224-y (PMC7745046; doi:10.1038/s41598-020-79224-y)
Supplement: Supplementary file 1 — Supplementary Information [file 41598_2020_79224_MOESM1_ESM.pdf]

Supplementary Information

Anomalous Blueshift of Aperture Resonance Enabled by the Loss of a Thin Film

Jisoo Kyoung

Department of Physics, Dankook University, 119 Dandae-ro, Dongnam-gu, Cheonan-si, Chungnam, 31116, Korea

kyoungjs@dankook.ac.kr

1. metal thickness effect

In our model, we assumed the metal as a perfect conductor. The perfect conductor, by definition, has infinitely large refractive index so that it reflects all the incident light regardless of its thickness. On the other hand, the real metal (Au or Ag) has finite refractive index or conductivity and thereby the transmission depends on the metal thickness. There have been many studies of terahertz transmittance depending on the thickness of gold film. For example, in the reference[1], the authors show that the THz transmission is less than 1/1,000 with only 30-nm-thick gold film. This means that a 70-nm-thick real metal film can be considered as a perfect conductor in the THz frequency range. The skin depth of the real metal is much larger than 70 nm because the wavelength of the THz wave is about 300 um to 3 mm. At the same time, the refractive index of the metal in this regime is about few hundreds (200~600)[2,3]. Once the light is passing through the surface of the metal, then it deeply penetrates. However, because of the very high refractive index, the amount of light passing through the metal surface is extremely small. Consequently, 70-nm-thick real metal film works as a perfect conductor.

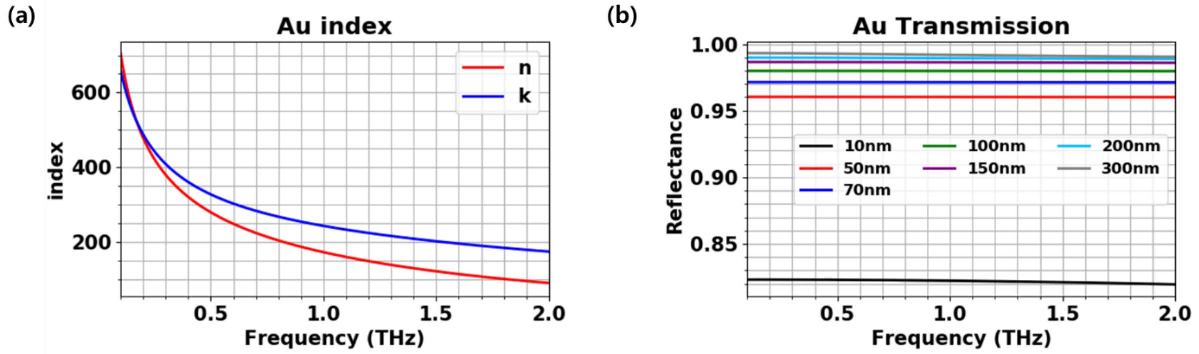

(a) Refractive index of Au film in THz regime (b) calculated transmission through Au film at various thicknesses.

2. thin thickness effect

We perform the calculation at 10 nm, 30 nm, and 100 nm film thicknesses. The calculation was performed under the following conditions: $w = 25$ μm, $l = 150$ μm, $h = 70$ nm, $p_x = 127$ μm, and $p_y = 180$ μm. Figure (a) shows real part dependency, while figure (b) represents imaginary part dependency. As clearly seen, red or blue shifts are observed in all cases even though the peak shift weakens as the film thickness is getting thinner. This means that such peak shifts are mainly due to the material properties of the thin film rather than the sample geometries.

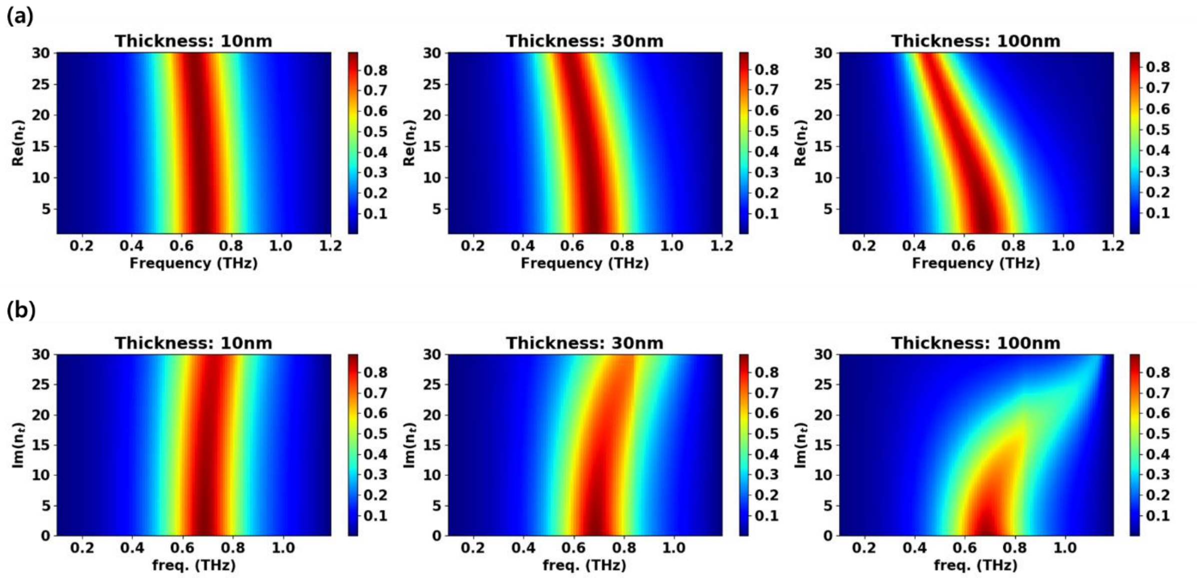

(a) Transmission as functions of frequency and $\mathrm{Re}(n_t)$ at 10 nm, 30 nm, and 100 nm thicknesses (b) Transmission as functions of frequency and $\mathrm{Im}(n_t)$ at 10 nm, 30 nm, and 100 nm thicknesses

3. top layer effect

For chemical or biological sensing applications, it would be more convenient to place the samples on top of the slot antennas rather than to insert between the slot antenna and the substrate. The top layer effect can be easily calculated by changing the superstrate index to the substrate index. By performing the calculation with same slot size and film thickness, we observed similar red and blue shifts according to change of the real and imaginary indices. The calculation was performed under the following conditions: $w = 25$ μm, $l = 150$ μm, $h = 70$ nm, $d = 240$ nm $p_x = 127$ μm, and $p_y = 180$ μm.

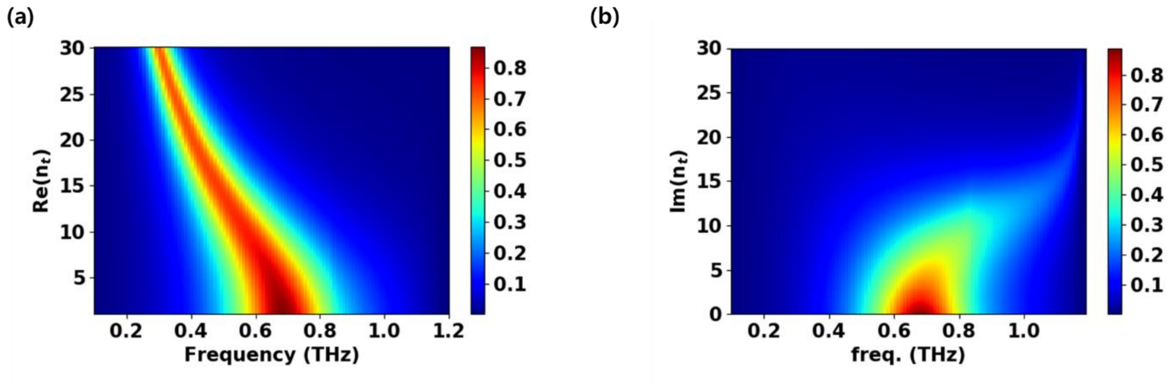

(a) Transmission as functions of frequency and Re(n_t) (b) Transmission as functions of frequency and Im(n_t)

4. comparison with Full-wave simulation

We performed the analytic calculation under the following conditions: $w = 25$ μm, $l = 150$ μm, $h = 70$ nm, $d = 300$ nm $p_x = 127$ μm, and $p_y = 180$ μm. The FDTD (finite difference time domain) simulation was done under the same condition except for the metal thickness. Since the metal thickness is too small compared to the wavelength, the metal is regarded as a perfect conductor with zero thickness to enhance the calculation efficiency. Figure (a) show the lossless thin film case with $n_t = 1.5, n_s = 2$, while the calculation results with dispersive lossy film is displayed in figure (b). As clearly seen, our model and the FDTD give consistent results in both cases.

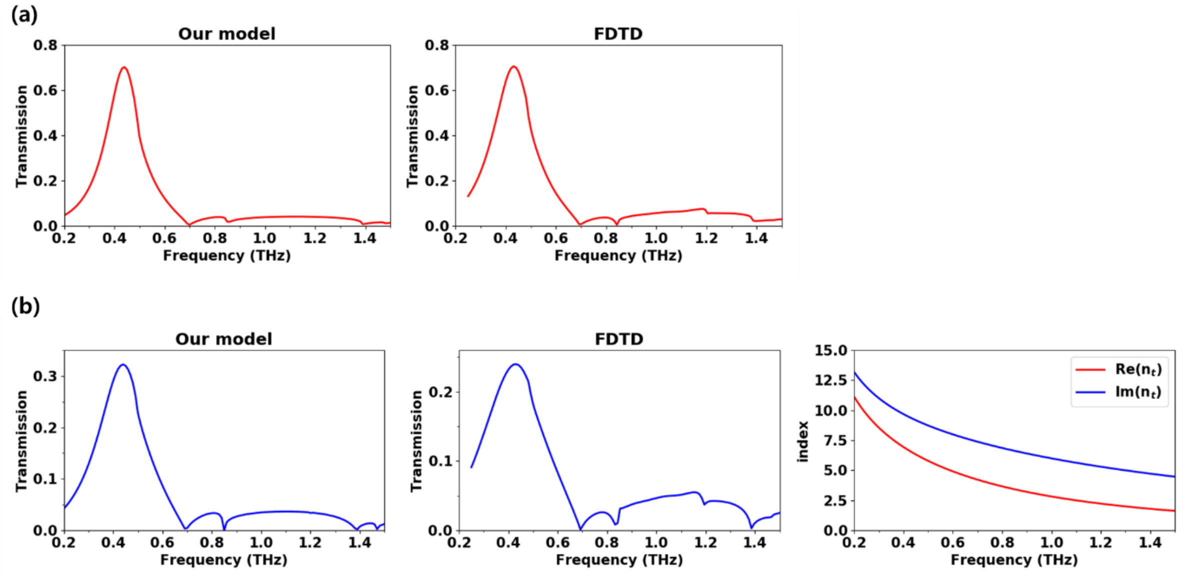

(a) left panel: our model, right panel: FDTD. $n_t = 1.5, n_s = 2$ (b) left panel: our model, center panel: FDTD, right panel: complex refractive index of a thin film.

1. Walther, M. *et al.* Terahertz conductivity of thin gold films at the metal-insulator percolation transition. *Phys. Rev. B* **76**, 125408 (2007).

2. Ordal, M. A., Bell, R. J., Alexander, R. W., Long, L. L. & Querry, M. R. Optical properties of Au, Ni, and Pb at submillimeter wavelengths. *Appl. Opt.* **26**, 744–752 (1987).

3. Yasuda, H. & Hosako, I. Measurement of Terahertz Refractive Index of Metal with Terahertz Time-Domain Spectroscopy. *Jpn. J. Appl. Phys.* **47**, 1632–1634 (2008).
